# Genomic features of *mecA*-harboring *Staphylococcus epidermidis* infecting an illegally traded rufous-bellied thrush (*T. rufiventris*)

**DOI:** 10.1007/s11259-026-11540-6

**Published:** 2026-09-28

**Authors:** Bianca Alvarenga Anti Pompeu, Gabriel Siqueira dos Santos, Ticiana Zwarg, Mayra Hespanhol Frediani, Amanda Haisi, João Pessoa Araújo, José Soares Ferreira Neto, Marcos Bryan Heinemann

**Affiliations:** 1 Departamento de Medicina Veterinária Preventiva e Saúde Animal, Faculdade de Medicina Veterinária e Zootecnia, Universidade de São Paulo, Av. Prof. Orlando Marques de Paiva, 87 - Butantã, São Paulo, 05508-270 Brazil; 2 Divisão da Fauna Silvestre, Prefeitura Municipal de São Paulo, Estrada de Perus, 300, São Paulo, 05276-11 Brazil; 3 Instituto de Biotecnologia, Universidade Estadual Paulista “Julio de Mesquita Filho”, Alameda das Tecomarias, Chácara Capão Bonito, Botucatu, 18607-440 Brazil

**Keywords:** Methicillin resistant, One Health, *Staphylococcus epidermidis*, Antibiotic resistance

## Abstract

*Staphylococcus epidermidis* is an opportunistic bacterium frequently involved in healthcare-associated infections and has become increasingly relevant in One Health when associated with methicillin resistance profile. Despite its significance in human medicine, the circulation of *S. epidermidis* in wildlife still poorly reported. In this study, we communicate the isolation and genomic characterization of a multidrug-resistant *S. epidermidis* Sequence Type 280 isolated from a skin lesion in an illegally traded wild rufous-bellied thrush (*Turdus rufiventris*). Antimicrobial susceptibility tests and genome sequencing revealed extensive multidrug resistance, including resistance to methicillin, related to the presence of antimicrobial resistance (*mecA*; *blaZ*; *aadD*; *aac(6′) − aph(2′′); fosB; erm(C)* and *rpoB* (D471E/I527M)). This finding provides compelling evidence for the global dissemination and ecological plasticity of this clone. Ultimately, the detection of methicillin-resistant *S. epidermidis* in a wild bird highlights the potential for urban environments to harbor and disseminate antimicrobial-resistant bacteria, highlighting the need to incorporate wildlife monitoring into One Health surveillance models.

## Background

The fragmentation of natural habitats caused by the rapid pace of urbanization has intensified the interaction between humans, domestic animals, and wildlife, facilitating the transmission of resistance genes because of the creation of ecological corridors (Doyle et al. 2025). In urbanized environments, synanthropic birds are considered vital epidemiological sentinels, as they reflect the antimicrobial selection pressure existing in shared ecosystems. The presence of antimicrobial-resistant strains in wild animals indicates that, while these animals may serve as sentinels, they also may play a role as mobile reservoirs that facilitate the dissemination of multidrug-resistant lineages across ecological boundaries (Vittecoq et al. 2016).

The Rufous-bellied Thrush (*Turdus rufiventris*) is a non-migratory resident species widely distributed across South America, occurring in southern Bolivia, Paraguay, Uruguay, central and coastal Argentina, and southern Brazil (Collar 2020). Although it inhabits a variety of natural environments, including gallery forests, Cerrado woodlands, and the Pampas, this species is highly tolerant of urbanized landscapes, which provide favorable conditions for colonization and population growth (Vazquez et al. 2024).

In human-animal-environment, methicillin-resistant non-aureus staphylococci species are becoming increasingly important as well(Nemeghaire et al. 2014; Schauer et al. 2021) as *Staphylococcus aureus*, which has been historically prioritized. The rise of these resistant strains, particularly *Staphylococcus epidermidis*, demonstrates the need for surveillance across diverse ecological niches, given that these are important reservoirs and vectors for bacterial resistance determinants (Dolejska and Literak 2019).

Among the non-aureus staphylococci, *S. epidermidis* has emerged as a significant opportunistic pathogen, distinguished by its genomic plasticity and ability to acquire several antimicrobial resistance (AMR) genes (Edslev et al. 2025b). While this bacterium is usually associated with infectious processes in humans, the circulation of high-risk lineages (Kellgren et al. 2024), such as Sequence Type 280 (ST280), in non-humans remains understudied.

This study reports the isolation and genomic characterization of an *S. epidermidis* ST280 strain with multiple antimicrobial resistance genes found in a skin lesion of an rufous-bellied thrush (*T. rufiventris*) at a wildlife rehabilitation center in São Paulo, Brazil. The identification of the resistance mechanisms presents in this strain, including genes conferring resistance to beta-lactams, aminoglycosides, rifampicin and macrolides, highlights the threat that bacterial clones pose to urban-wildlife interactions and underscores the urgent need for genomic surveillance in free-living species.

## Case presentation

On September 2024, an illegally traded rufous-bellied thrush *(Turdus rufiventris)* was rescued by the municipal guard and admitted in a wild animal medical care and rehabilitation center in São Paulo State, Brazil. The animal presented skin lesions in cephalic region and a swab sample was collected for microbiological diagnostic. The swab was later streaked on Blood Agar (5% v/v) and incubated at 37 °C overnight. White circular colonies were identified as *Staphylococcus epidermidis* by MALDI-TOF (scores 2.36 and 2.32). After unsuccessful treatment with cefalexin (0.08 mg/g of body weight) and ketoconazole (0.02 mg/g of body), and worsening of its clinical status, the animal was euthanized.

For the tested strain designated as SL01, antimicrobial resistance profile was assessed by the minimum inhibitory concentration (MIC) values of the antimicrobials, which were determined by the broth microdilution method and interpreted according to the Clinical and Laboratory Standards Institute (CLSI) M100-S36 guidelines (CLSI, 2026). The antimicrobial concentration range tested is presented in Table 1.

The genomic library was prepared with 300 ng of dsDNA using a modified protocol of Illumina COVIDSeq (Illumina Inc., San Diego, USA), starting from the tagmentation step, and all subsequent procedures were performed according to the manufacturer’s instructions. Library quality was assessed using the TapeStation High Sensitivity D1000 ScreenTape Assay (Agilent Technologies, Santa Clara, USA). The whole genome sequencing of the SL01 isolate (Genbank Accession: JBWXTE000000000) was performed using the MiSeq i100 platform (Illumina Inc., San Diego, CA) with 150 bp single-end reads. The quality of the reads was assessed by FastQC (v.0.12.1) (https://github.com/s-andrews/FastQC*)*, and the reads were trimmed using Trimmomatic (v.0.39) (https://github.com/usadellab/Trimmomatic*).*

Unicycler (v.0.5.1) (https://github.com/rrwick/Unicycler) was used to assemble the draft genome, which was later annotated with Prokka (v.1.14.6) (https://github.com/tseemann/prokka). Genomic data were deposited in GenBank (https://www.ncbi.nlm.nih.gov/genbank/).

Antimicrobial resistance genes were screened by the ABRicate v1.0.1 platform (https://github.com/tseemann/abricate) with ResFinder 4.6.0 (http://genepi.food.dtu.dk/resfinder*).* Later, bacterial typing tools were used to identify Multi-Locus Sequence Typing (MLST) (MLST v.2.0) (https://github.com/tseemann/mlst*).*

All *Staphylococcus epidermidis* genomes available in NCBI’s Genome platform (https://www.ncbi.nlm.nih.gov/home/genomes/) were compared to SL01 genome using FastANI v1.32 (https://github.com/ParBLiSS/FastANI). The 50 genomes with the highest ANI were selected for phylogenetic analysis. The genome annotation was performed using Prokka v1.14.6 (https://github.com/tseemann/prokka), the core orthologous genes of these genomes were concatenated and aligned using Roary (v3.13.0) (https://github.com/sanger-pathogens/Roary), and a maximum likelihood phylogenetic tree was constructed with IQ-TREE (v2.4.0) (https://iqtree.github.io/) (ultrafast bootstrap 1000 x). For comparative analysis, a phylogenomic tree and a heatmap with antimicrobial resistance genes were constructed with R software (R Core Team 2021) by RStudio (RStudio Team 2020) using the ggplot2 (Wickham 2009) and ggtreeExtra (Xu et al. 2021) packages for graphing.

The isolate obtained from the skin lesion found in a rufous-bellied thrush was identified as *Staphylococcus epidermidis*. The Minimum Inhibitory Concentration (MIC) test found that the SL01 strain exhibited resistance to multiple antimicrobial classes (Table 1). The isolate showed elevated resistance to beta-lactams, specifically penicillin (MIC = 0.5 µg/mL) and oxacillin (MIC = 1 µg/mL), as well as to gentamicin (MIC = 16 µg/mL). Additionally, resistance was observed for rifampicin (MIC = 8 µg/mL), and clindamycin (MIC = 8 µg/mL).

**Table 1:** Antimicrobial susceptibility profile and corresponding antimicrobial resistance genes for *Staphylococcus epidermidis* SL01 strain results and respective antimicrobial resistance genes associated

Antimicrobial	Minimum inhibitory concentration (µg/mL)	Minimal concentration tested (µg/mL)	Maximum concentration tested (µg/mL)	Result (CLSI, 2026)	Antimicrobial resistance gene related
Oxacillin	1	0.25	2	Resistant	*mecA*
Penicillin	0.5	0.0625	1	Resistant	*mecA; blaZ*
Gentamicin	16	2	32	Resistant	*aadD; aac(6′) − aph(2′′)*
Chloramphenicol	2	2	64	Susceptible	*-*
Ciprofloxacin	1	0.5	8	Susceptible	*-*
Clindamycin	8	0.25	8	Resistant	*erm(C)*
Rifampicin	8	0.5	8	Resistant	*rpoB* (D471E/I527M)
Sulfamethoxazole/trimethoprim	38/2	19/1	152/8	Susceptible	*-*
Vancomycin	2	2	64	Susceptible	-
Tetracycline	2	2	32	Susceptible	-

The multi-locus sequencing typing analysis revealed that the *S. epidermidis* isolate belonged to ST280. Annotation analysis revealed D471E/I527M substitutions in subunit of bacterial beta-subunit RNA polymerase. The ABRicate screening identified the presence of genes conferring resistance to β-lactams (*mecA*; *blaZ*), aminoglycosides (*aadD*; *aac(6´)-aph(2′′)*), fosfomycin (*fosB*), and macrolides (*erm(C)*).

**Fig. 1:**
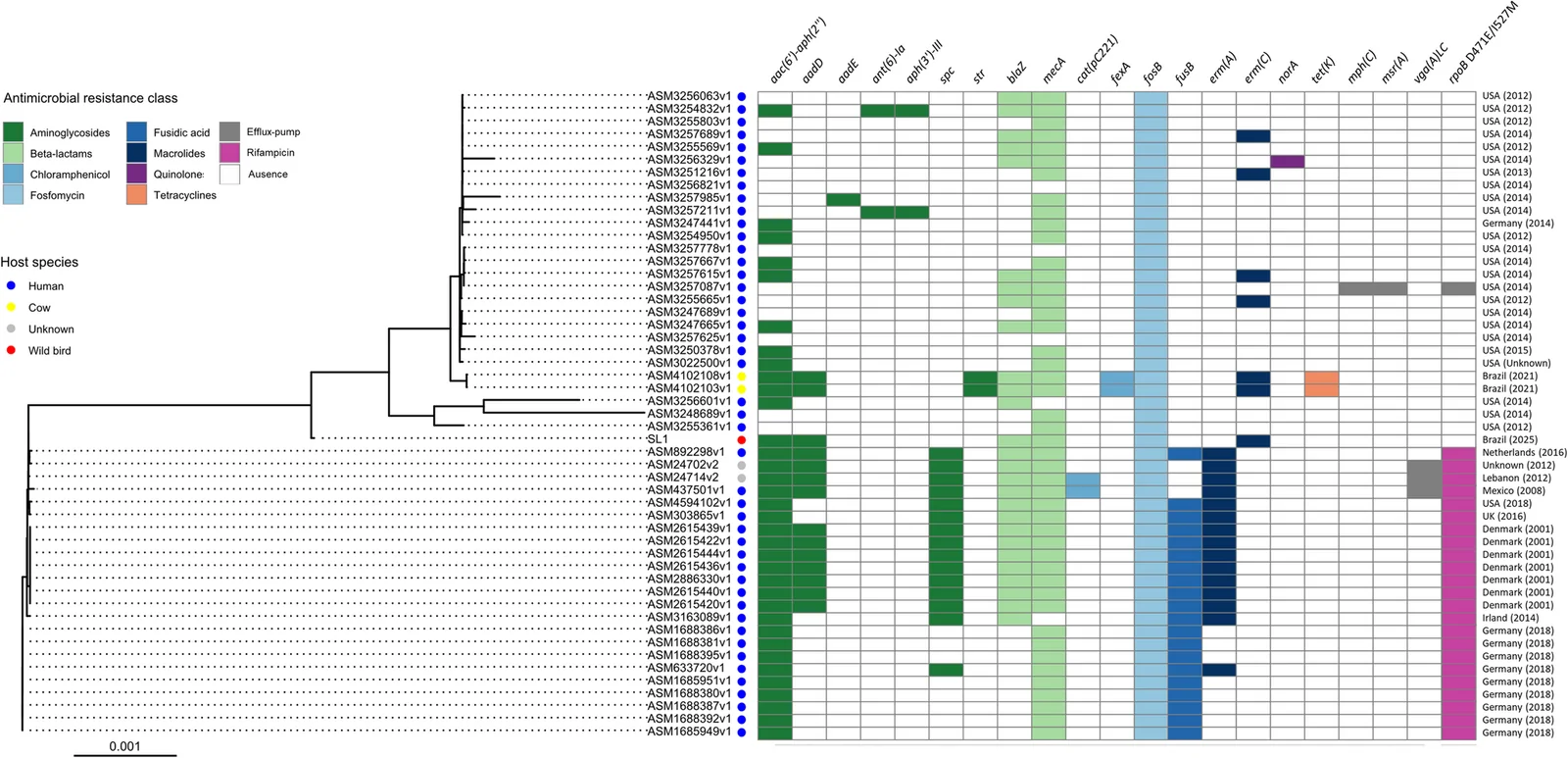
Phylogenetic and comparative genomic analysis of *Staphylococcus epidermidis* isolates. Maximum likelihood phylogenetic tree based on the core genome (concatenated orthologous genes) of *S. epidermidis* SL01 and the 50 genomes with the highest average nucleotide identity (ANI) from the NCBI database. The tree was constructed using IQ-TREE with 1,000 ultrafast bootstrap replicates. The associated heatmap displays the presence (filled) and absence (empty) of antimicrobial resistance genes identified via Prokka annotation. The analysis was visualized using R software with *ggplot2* and *ggtreeExtra* packages

Phylogenomic and epidemiological analysis reveals that *S. epidermidis* ST280 is distributed worldwide. Most of *S. epidermidis* strains (90.2%; 46/51) were reported in humans and showed antimicrobial resistance encoding genes for aminoglycosides (74.5%; 38/51), beta-lactams (94.1%; 48/51) and fosfomycin (100%) (Fig. 1). All studied samples carried the fosfomycin resistance gene *fosB*, 46 samples (90.2%; 46/51) were positive for *mecA* and 26 (51.0%; 26/51) positive for *blaZ.* SL01 isolate did not cluster with any specific pattern of strains (Fig. 1).

## Discussion

In this study we reported the isolation of a multidrug-resistant (MDR) *Staphylococcus epidermidis* ST280 from a skin lesion in a rufous-bellied thrush (*Turdus rufiventris*). This finding is particularly significant as *S. epidermidis* is a well-documented opportunistic pathogen associated with hospital environments and healthcare-associated infections in humans (Rupp 2014; Dong et al. 2018; Chabi and Momtaz 2019; Martínez-Santos et al. 2022; Demiselle et al. 2023) and demonstrates the strong impact that anthropogenic pressure has on wildlife health.

SL01 isolate genomic profile reveals a strict relation to the animal outcome, considering its difficulty in its pharmacological treatment. The presence of the *mecA* and *blaZ* genes are related to the lack of susceptibility to beta-lactams. In addition, the identification of *erm(C)*, *aadD*, and *aac(6’)-aph(2’’)* genes highlights the strain’s ability to resist multiple classes of antimicrobials, including macrolides and aminoglycosides. In a healthcare context, the multiple antimicrobial resistance demonstrated in this strain shows a clinical challenge in treatment (Kalın et al. 2023), which may have contributed to treatment failure.

The identification of the SL01 isolate in a wild bird in Brazil provides compelling evidence for the global dissemination of ST280 *S. epidermidis* lineages (Miragaia et al. 2008; Lee et al. 2018). This finding suggests that wild birds may act as environmental sentinels, bringing together clinical settings, livestock production, and natural ecosystems (Dalazen et al. 2023; Mbuthia and Hoza 2025). The genomic resemblance between SL01 and isolates recovered over a decade ago in different countries (Fig. 1) indicates a successful clone with remarkable ecological plasticity (Pitout and DeVinney 2017). In addition, this intercontinental and multi-host connectivity points towards human-to-animal transmission, a condition where human-associated pathogens spill over into wildlife because of environmental contamination (Ellwanger et al. 2022). From a One Health perspective, our results highlight that the virulence potential of *S. epidermidis* is not confined to hospitals, but is actively circulating across the human-animal-environment interface, regardless of geographical borders (Persson Waller et al. 2025).

To the best of our knowledge, this is the first case linked to an MDR *S. epidermidis* infection to a wild animal’s demise, marking a significant turning point in wildlife pathology. The skin lesion in *T. rufiventris* with a methicillin-resistant *S. epidermidis* shows that these human-associated lineages can transcend species boundaries and may exhibit pathogenic potential in susceptible wild hosts, in contrast to earlier research that classified non-aureus staphylococci in wildlife as commensals or asymptomatic carriage (González-Martín et al. 2020). Since fosfomycin and rifampicin are frequently used as a last resort for MDR staphylococcal infections (Dinh et al. 2012; Bodmann et al. 2025), the presence of *rpoB* (D471E/I527M) mutation, substitution frequently associated with rifampicin resistance in *Staphylococcus epidermidis* (Edslev et al. 2025a) and fosfomycin resistance (*fosB*) further complicates the resistance landscape. Therefore, these findings support the need to incorporate wildlife monitoring into One Health models (Barroso et al. 2023).

The detection of a synanthropic sentinel harboring an MDR clone such as ST280 provides a stark indicator of “antimicrobial pollution” in urban centers such as São Paulo (Laborda et al. 2022). Future surveillance must focus not only on the presence of these bacteria but also on their pathological impact, as the transmission of hospital-associated resistance determinants to wildlife may lead to localized biodiversity losses and create permanent environmental reservoirs of high-risk pathogens. Since the spread of hospital-associated resistance determinants to wildlife may result in localized biodiversity losses and establish permanent environmental reservoirs of high-risk pathogens (Laborda et al. 2022; Silva et al. 2025), future surveillance must concentrate on both the presence of these bacteria and their pathological impact. 

## Data Availability

No datasets were generated or analysed during the current study.

## References

[CR1] Barroso P, Relimpio D, Zearra JA et al (2023) Using integrated wildlife monitoring to prevent future pandemics through one health approach. One Health 16:100479. 10.1016/j.onehlt.2022.100479 PMC9806683 36600947

[CR2] Bodmann K-F, Hagel S, Oliva A et al (2025) Real-World Use, Effectiveness, and Safety of Intravenous Fosfomycin: The FORTRESS Study. Infect Dis Ther 14:765–791. 10.1007/s40121-025-01125-2 PMC11993532 40106180

[CR3] Chabi R, Momtaz H (2019) Virulence factors and antibiotic resistance properties of the Staphylococcus epidermidis strains isolated from hospital infections in Ahvaz, Iran. Trop Med Health 47:56. 10.1186/s41182-019-0180-7 PMC6896349 31844416

[CR4] Clinical and Laboratory Standards Institute (2026) Performance standards for antimicrobial susceptibility. M100-S36 edn.

[CR5] Collar N (2020) Rufous-bellied thrush (*Turdus rufiventris*). In: del Hoyo J, Elliott A, Sargatal J et al (eds) Birds of the world. Cornell Lab Ornithol

[CR6] Dalazen G, Fuentes-Castillo D, Pedroso LG et al (2023) CTX-M-producing Escherichia coli ST602 carrying a wide resistome in South American wild birds: Another pandemic clone of One Health concern. One Health 17:100586. 10.1016/j.onehlt.2023.100586 PMC10320584 37415721

[CR7] Demiselle J, Meyer P, Lavigne T et al (2023) Staphylococcus epidermidis bloodstream infections are a cause of septic shock in intensive care unit patients. Int J Infect Dis 135:45–48. 10.1016/j.ijid.2023.07.014 37517512

[CR8] Dinh A, Salomon J, Bru JP, Bernard L (2012) Fosfomycin: Efficacy against infections caused by multidrug-resistant bacteria. Scand J Infect Dis 44:182–189. 10.3109/00365548.2011.616221 22176655

[CR9] Dolejska M, Literak I (2019) Wildlife Is overlooked in the epidemiology of medically important antibiotic-resistant bacteria. Antimicrob Agents Chemother vol 63. 10.1128/AAC.01167-19 PMC6658798 31209001

[CR10] Dong Y, Speer CP, Glaser K (2018) Beyond sepsis: *Staphylococcus epidermidis* is an underestimated but significant contributor to neonatal morbidity. Virulence 9:621–633. 10.1080/21505594.2017.1419117 PMC5955464 29405832

[CR11] Doyle C, Wall K, Fanning S, McMahon BJ (2025) Making sense of sentinels: wildlife as the One Health bridge for environmental antimicrobial resistance surveillance. J Appl Microbiol vol 136. 10.1093/jambio/lxaf017 39805713

[CR12] Edslev SM, Aarris M, Nielsen KL et al (2025a) *rpoB* mutations and their association with rifampicin resistance in clinical *Staphylococcus epidermidis*. J Antimicrob Chemother 80:1067–1071. 10.1093/jac/dkaf035 PMC11962384 39913260

[CR13] Edslev SM, Laurent F, Månsson E et al (2025b) Antibiotic resistance and population structure of *Staphylococcus epidermidis* from prosthetic joint infections in Sweden and France. J Antimicrob Chemother 80:2007–2015. 10.1093/jac/dkaf166 PMC12209789 40459065

[CR14] Ellwanger JH, Fearnside PM, Ziliotto M et al (2022) Synthesizing the connections between environmental disturbances and zoonotic spillover. Acad Bras Cienc vol 94. 10.1590/0001-3765202220211530 36169531

[CR15] González-Martín M, Corbera JA, Suárez-Bonnet A, Tejedor-Junco MT (2020) Virulence factors in coagulase-positive staphylococci of veterinary interest other than *Staphylococcus aureus*. Veterinary Q 40:118–131. 10.1080/01652176.2020.1748253 PMC7178840 32223696

[CR16] Kalın G, Alp E, Chouaikhi A, Roger C (2023) Antimicrobial Multidrug Resistance: Clinical Implications for Infection Management in Critically Ill Patients. Microorganisms 11:2575. 10.3390/microorganisms11102575 PMC10609422 37894233

[CR17] Kellgren T, Dwibedi C, Widerström M et al (2024) Completed genome and emergence scenario of the multidrug-resistant nosocomial pathogen Staphylococcus epidermidis ST215. BMC Microbiol 24:215. 10.1186/s12866-024-03367-5 PMC11186124 38890594

[CR18] Laborda P, Sanz-García F, Ochoa-Sánchez LE et al (2022) Wildlife and antibiotic resistance. Front Cell Infect Microbiol vol 12. 10.3389/fcimb.2022.873989 PMC9130706 35646736

[CR19] Lee JYH, Monk IR, Gonçalves da Silva A et al (2018) Global spread of three multidrug-resistant lineages of Staphylococcus epidermidis. Nat Microbiol 3:1175–1185. 10.1038/s41564-018-0230-7 PMC6660648 30177740

[CR20] Martínez-Santos VI, Torres-Añorve DA, Echániz-Aviles G et al (2022) Characterization of *Staphylococcus epidermidis* clinical isolates from hospitalized patients with bloodstream infection obtained in two time periods. PeerJ 10:e14030. 10.7717/peerj.14030 PMC9541613 36213498

[CR21] Mbuthia CW, Hoza AS (2025) Wild birds as potential reservoirs of antimicrobial-resistant *Escherichia coli*: a systematic review. Front Microbiol vol 16. 10.3389/fmicb.2025.1615826 PMC12450974 40988852

[CR22] Miragaia M, Carriço JA, Thomas JC et al (2008) Comparison of Molecular Typing Methods for Characterization of *Staphylococcus epidermidis*: Proposal for Clone Definition. J Clin Microbiol 46:118–129. 10.1128/JCM.01685-07 PMC2224289 17989196

[CR23] Nemeghaire S, Argudín MA, Feßler AT et al (2014) The ecological importance of the Staphylococcus sciuri species group as a reservoir for resistance and virulence genes. Vet Microbiol 171:342–356. 10.1016/j.vetmic.2014.02.005 24629775

[CR24] Persson Waller K, Myrenås M, Kim H et al (2025) Dissemination of *Staphylococcus epidermidis* in Swedish bovine dairy herds: minimal overlap with human isolates. Front Microbiol vol 16. 10.3389/fmicb.2025.1512461 PMC11849392 39996079

[CR25] Pitout JDD, DeVinney R (2017) *Escherichia coli* ST131: a multidrug-resistant clone primed for global domination. F1000Res 6:195. 10.12688/f1000research.10609.1 PMC5333602 28344773

[CR26] R Core Team (2021) R: a language and environment for statistical computing.

[CR27] RStudio T (2020) RStudio: integrated development for R.

[CR28] Rupp ME (2014) Clinical characteristics of infections in humans due to *Staphylococcus epidermidis*. pp 1–16 10.1007/978-1-62703-736-5_1 24222451

[CR29] Schauer B, Szostak MP, Ehricht R et al (2021) Diversity of methicillin-resistant coagulase-negative Staphylococcus spp. and methicillin-resistant Mammaliicoccus spp. isolated from ruminants and New World camelids. Vet Microbiol 254:109005. 10.1016/j.vetmic.2021.109005 33582485

[CR30] Silva V, Araújo S, Caniça M et al (2025) Caught in the ESKAPE: Wildlife as Key Players in the Ecology of Resistant Pathogens in a One Health Context. Divers (Basel) 17:220. 10.3390/d17040220

[CR31] Vazquez MS, La Sala LF, Scorolli AL et al (2024) Pushing the boundaries: actual and potential distribution of thrushes expanding their ranges in South America. Sci Rep 14:17587. 10.1038/s41598-024-68611-4 PMC11289275 39080378

[CR32] Vittecoq M, Godreuil S, Prugnolle F et al (2016) Antimicrobial resistance in wildlife. J Appl Ecol 53:519–529. 10.1111/1365-2664.12596

[CR33] Wickham H (2009) ggplot2. Springer New York, New York, NY

[CR34] Xu S, Dai Z, Guo P et al (2021) ggtreeExtra: Compact Visualization of Richly Annotated Phylogenetic Data. Mol Biol Evol 38:4039–4042. 10.1093/molbev/msab166 PMC8382893 34097064

